# Liver transplant patient with in-transit squamous cell carcinoma treated with talimogene laherparepvec

**DOI:** 10.1016/j.jdcr.2023.07.033

**Published:** 2023-08-09

**Authors:** Jamie Lebhar, Jennifer Jacobs, Khushnood Faraz, April K.S. Salama, Paul J. Mosca

**Affiliations:** a Duke University School of Medicine, Durham, North Carolina; b Division of Medical Oncology, Duke University Health System, Durham, North Carolina; c Division of Surgical Oncology, Duke University Health System, Durham, North Carolina

**Keywords:** intralesional, in-transit, liver transplant, squamous cell carcinoma, talimogene Laherparepvec, T-VEC

## Introduction

Nonmelanoma skin cancers (NMSCs), especially cutaneous squamous cell carcinomas (cSCCs), are extremely common in immunosuppressed patients.^1^ Most solid organ transplant recipients (SOTRs) are at markedly elevated risk for developing cutaneous malignancies due to the lifelong immunosuppression required to avoid allogenic graft reaction. While anti PD-1 based immunotherapy (IO) represents the standard of care for unresectable cSCC, the limited efficacy and concern for inducing graft rejection limits its use in SOTR.^2^ Recently, novel local therapies for the treatment of advanced or recurrent cutaneous malignancies have received significant attention. Talimogene laherparepvec (T-VEC) is the first intralesional oncolytic virus therapy with Food and Drug Administration (FDA) approval to treat advanced-stage melanoma. Although T-VEC is not approved to treat NMSC and is contraindicated in immunosuppressed patients, there are some SOTRs for whom it is among the safest and most appropriate options.^3^^,^^4^ This report describes the treatment of SOTR patient with recurrent cSCC who underwent successful treatment with T-VEC as part of a multi-modality, limb-sparing approach to controlling the disease.

## Case report

A 71-year-old man with a history of Evan’s syndrome, orthotopic liver transplantation in 2016, and recurrent cSCC of the left forearm presented to Duke Cancer Institute for management of an additional recurrent cSCC. Four years prior to presentation, he underwent a whole, deceased-donor liver allotransplantation due to end stage liver disease secondary to autoimmune hepatitis and primary sclerosing cholangitis. Postoperative complications included biliary enteric anastomotic stricture requiring reoperation, as well as subsequent infections. The following year he had an acute rejection episode requiring pulsed IV steroids and an increased dosing of immunosuppressants. At presentation for his most recent recurrence of cSCC, his immunosuppression regimen consisted of prednisone 5 mg once daily, tacrolimus 2 mg once in the morning and 1 mg nightly, and mycophenolate 250 mg twice daily. Mycophenolate was later discontinued and replaced with sirolimus 2 mg daily due to his recurrent cSCC.

Two years post-transplantation, the patient noticed a suspicious lesion on his left arm and was diagnosed with moderately differentiated cSCC. Subsequently, over a 2-year period, he underwent 4 Mohs micrographic surgeries on his left proximal forearm and elbow region, skin grafting, and from lack of disease control, underwent adjuvant external beam radiation therapy (RT). Two years after initial cSCC diagnosis, he developed recurrence as in transit metastasis on his left arm, with 3 punch biopsies of separate lesions showing metastatic, moderately to poorly differentiated cSCC involving the deep dermis with areas of ulceration and lymphovascular invasion. PET CT imaging showed hypermetabolic skin thickening about the left elbow and forearm with lesions ranging from 5 to 23 mm. Additional surgery was unfavorable given the pattern of disease recurrence. Treatment consideration was given to regional chemotherapy with isolated limb infusion, but given the risk of hepatotoxicity and limb-threatening toxicity, this option was less favorable. Systemic immunotherapy (IO) with an anti-PD-1 antibody was reserved as a last resort due to the risk of life-threatening organ rejection and limited efficacy in immunosuppressed patients.^5^^,^^6^ EGFR inhibitors were disfavored due to the risk of toxicity. Finally, intralesional treatment with the oncolytic viral agent, T-VEC, was discussed. There was concern for toxicity, particularly for a disseminated herpesvirus infection, but based on literature review and discussions, this risk was deemed as low.^7^^,^^8^ T-VEC treatment was offered to and elected by the patient.

**Fig 1:**
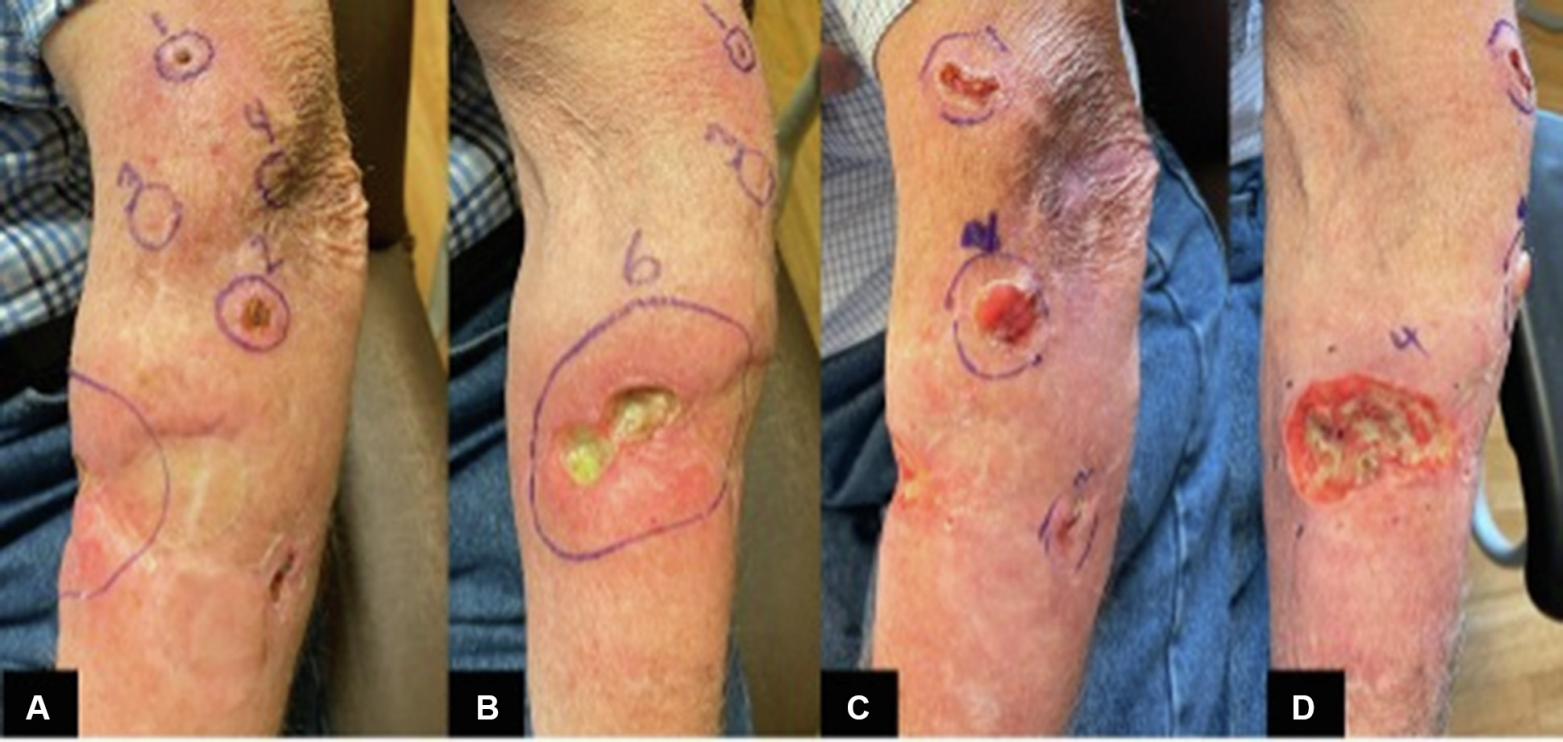
*Left forearm* notable for multiple in-transit lesions of various sizes. Images (**A** and **B**), were taken on T-VEC treatment 2 (day 21) and images (**C** and **D**), on treatment 16 (day 217).

Due to the proposed off-label use of T-VEC, approvals were obtained through Duke Health IRB and regulatory oversight processes, and an individual patient FDA IND application. The drug was provided by Amgen free of charge through an expanded access program. Once treatment was initiated, 2 adjacent lesions had coalesced to form a large, ulcerated lesion (Fig 1).

The patient was treated with T-VEC therapy in accordance with FDA prescribing, using an initial concentration of 10^6^ pfu/ml followed by 10^8^ pfu/ml for all subsequent treatments (Table I). T-VEC treatment consisted of 32 cycles every 2 wk over a 15-month period (Table I). After complete elimination of all lesions with T-VEC and 1 surgical excision, a new focus of hypermetabolic activity was visualized at the ulnar aspect of the forearm via PET imaging. Fine needle aspiration of the palpable subcutaneous nodule revealed a new in-transit lesion of cSCC. Notably, because this lesion progressed rapidly and did not show early signs of responsiveness to T-VEC, the treatment was terminated and definitive external beam RT was administered. Treatment consisted of 6 fractions of 600 cGy for a total dose of 3,600 cGy using en face electrons at 6 MeV. Following RT, the patient has continued surveillance with physical exam and cross-sectional imaging. Thirteen months post T-VEC discontinuation, imaging revealed no evidence of active disease.

**Table I:** Talimogene laherparepvec treatment for the patient’s left forearm squamous cell carcinoma lesions that occurred every 2 weeks for a period of 15 months

T-VEC Treatment number	Days from T-VEC initiation	T-VEC Dose (pfu/mL)	Number of sites	Notable progress
1	0	10^6^	6	CT of left upper extremity with contrast without evidence for nodal or distant disease.
2	21	10^8^	5	
3	35	10^8^	5	Erythema surrounding largest lesion improved.
4	49	10^8^	5	Largest lesion with ulceration measuring 4.5 cm longest axis and 0.7 cm deep. Surrounding erythema has nearly resolved.
5	63	10^8^	5	Whole body PET CT imaging without evidence for nodal or distant disease.
6	77	10^8^	5	
7	91	10^8^	4	Repeat CT of left upper extremity with contrast without evidence for distant disease. Swelling and induration surrounding forearm ulceration has been stable and is mild. Patient labs
8	105	10^8^	3	Noted new friability of a lesion.
9	119	10^8^	3	
10	133	10^8^	3	
11	147	10^8^	3	Largest lesion with ulceration measuring 5.5 cm in longest axis. Friability present in second lesion measuring 1.8 cm.
12	161	10^8^	2	Repeat whole body PET CT imaging without evidence for nodal or distant disease.
13	175	10^8^	3	Lesions continuing to fill in and contract. One lesion resolved.
14	189	10^8^	4	Lesions noted to have blood oozing.
15	203	10^8^	4	
16	217	10^8^	4	
17	231	10^8^	3	Due to resistance to treatment, one lesion was excised.
18	245	10^8^	6	
19	259	10^8^	4	Repeat whole body PET CT revealed slightly decreased activity of left forearm lesions and no distant disease.
20	273	10^8^	3	Significant clinical response to treatment and no new definitive lesions.
21	287	10^8^	3	
22	301	10^8^	3	
23	322	10^8^	2	
24	336	10^8^	2	
25	350	10^8^	3	
26	364	10^8^	3	Repeat whole body PET CT showed new focus of hypermetabolic activity ulnar aspect of left forearm suspicious for disease recurrence.
27	378	10^8^	2	Fine needle aspiration of left arm nodule performed and was nondiagnostic. Continued with treating the site as another focus of SCC.
28	399	10^8^	2	
29	413	10^8^	2	One evident remaining lesion has completely filled in and re-epithelialized. Subcutaneous ulnar lesion stable in size.
30	427	10^8^	2	
31	441	10^8^	2	Repeat fine needle aspiration of arm lesion revealed SCC.
32	455	10^8^	2	Subcutaneous nodule grew, now measuring 2.5 cm. Repeat whole body PET CT showed increasing hypermetabolic activity in soft tissues of left arm, concerning for SCC recurrence.

He tolerated the treatment well overall. Of note, between T-VEC treatment 6 and 7, the patient was thrombocytopenic with counts ranging from 20,000 to 80,000. Attribution to T-VEC treatment was felt to be unlikely. He did not experience any bleeding complications during this time and was managed medically with eltrombopag. Platelet counts returned to normal range within 2 months of initiating treatment, and no other side effects have been reported. Importantly, he experienced no clinically evident herpesvirus infection.^7^

## Conclusions

**Fig 2:**
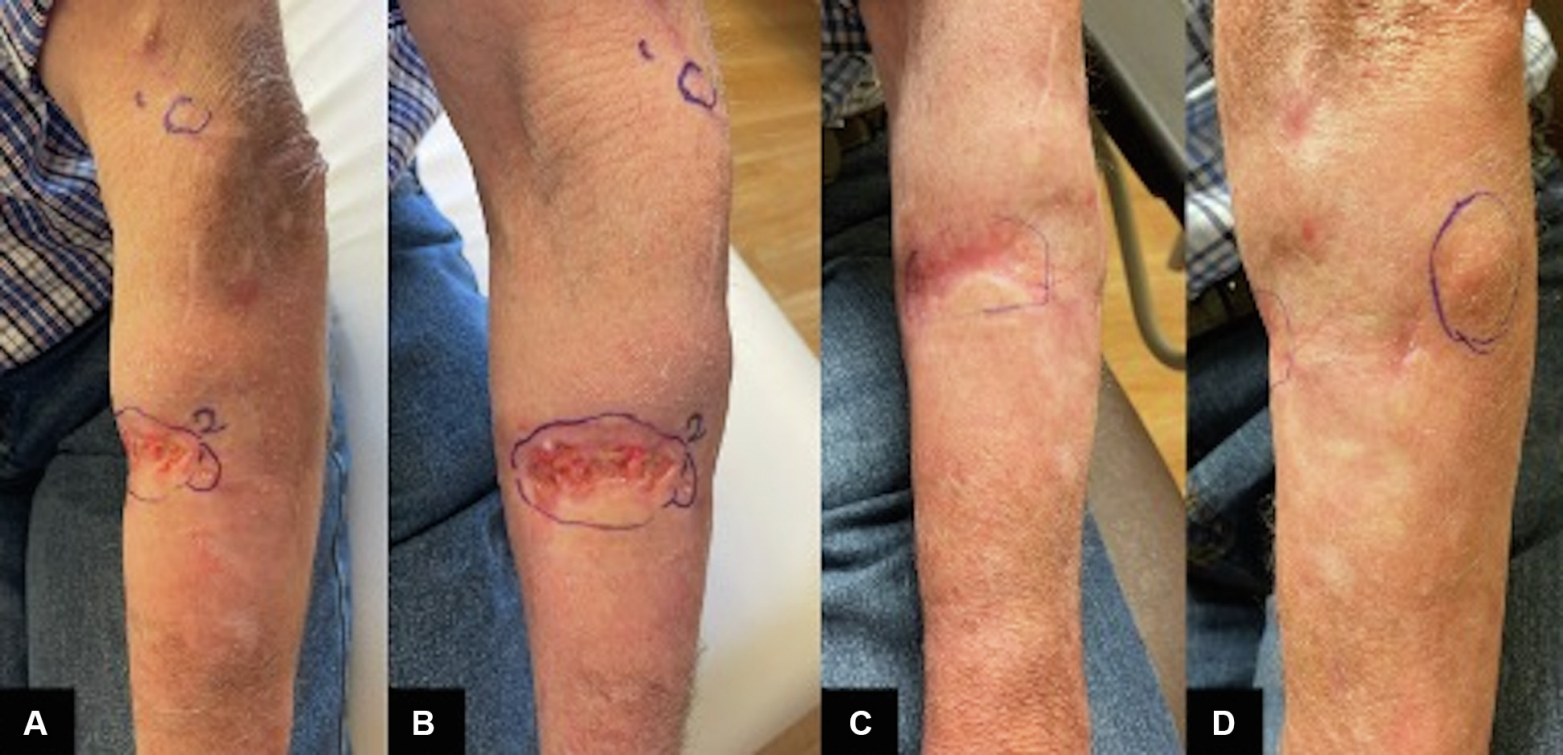
*Left forearm* notable for multiple in-transit lesions of various sizes. Images (**A** and **B**), were taken on T-VEC treatment 24 (day 336) and images (**C** and **D**), on treatment 32 (day 455). The images depict a significant healing of lesions with re-epithelialization. Image (**D**), illustrates the new subcutaneous nodule measuring 2.5 cm that became noticeable just as the last of the prior lesions had completely resolved.

Intralesional oncolytic viral therapy has potential clinical utility for patients with recurrent cSCC following SOTR and may be used as a multimodal treatment strategy. This case demonstrates the use of T-VEC therapy for the treatment of cSCC in a patient with complex medical history including liver transplantation, graft rejection, and autoimmune disease. The patient underwent 32 intralesional T-VEC treatments which helped eradicate his in-transit cSCC (Figs 1 and 2). The disease ultimately progressed, revealing a new solitary nodule, which completely resolved with RT. He has remained without evidence of recurrent disease 9 months after completion of RT. This case highlights the safety and utility of T-VEC to treat recurrent in-transit cSCC in a SOTR. Ongoing clinical trials, including the ARTACUS trial (NCT04349436), a phase Ib/II study evaluating intralesional RP1, a next-generation herpesvirus-based oncolytic therapy, in SOTR with advanced cutaneous malignancies, could establish this as a standard treatment strategy in the future.^9^ Further investigation is warranted to elucidate the utility of T-VEC in treating cutaneous cSCC in SOTR.

## Conflicts of interest

Salama received research funding (paid to institution) from Ascentage, Bristol Myers Squibb, Ideaya, Immunocore, Merck, Olatec Therapeutics, Regeneron, Replimune, and Seagen and is consultant or is in advisory role for Bristol Myers Squibb, Iovance, Regeneron, Novartis, and Pfizer.
